# Glycosylated Flavonoid Compounds as Potent CYP121 Inhibitors of *Mycobacterium tuberculosis*

**DOI:** 10.3390/biom12101356

**Published:** 2022-09-23

**Authors:** Leena Hussein Bajrai, Aiah M. Khateb, Maha M. Alawi, Hashim R. Felemban, Anees A. Sindi, Vivek Dhar Dwivedi, Esam Ibraheem Azhar

**Affiliations:** 1 Special Infectious Agents Unit-BSL3, King Fahd Medical Research Center, King Abdulaziz University, Jeddah 21362, Saudi Arabia; 2 Biochemistry Department, Faculty of Sciences, King Abdulaziz University, Jeddah 21362, Saudi Arabia; 3 Department of Medical Laboratory Technology, Faculty of Applied Medical Sciences, Taibah University, Madinah 42353, Saudi Arabia; 4 Department of Medical Microbiology and Parasitology, Faculty of Medicine, King Abdulaziz University, Jeddah 21589, Saudi Arabia; 5 Infection Control & Environmental Health Unit, King Abdulaziz University Hospital, King Abdulaziz University, Jeddah 21589, Saudi Arabia; 6 Medical Laboratory Sciences Department, Faculty of Applied Medical Sciences, King Abdulaziz University, Jeddah 21362, Saudi Arabia; 7 Department of Anesthesia and Critical Care, Faculty of Medicine, King Abdulaziz University, Jeddah 21589, Saudi Arabia; 8 Bioinformatics Research Division, Quanta Calculus Pvt. Ltd., Greater Noida 201310, India; 9 Institute of Advanced Materials, IAAM, 59053 Ulrika, Sweden

**Keywords:** CYP121, *Mycobacterium tuberculosis*, protein-ligand interaction, docking, molecular dynamic simulation

## Abstract

Due to the concerning rise in the number of multiple- and prolonged-drug-resistant (MDR and XDR) *Mycobacterium tuberculosis* (Mtb) strains, unprecedented demand has been created to design and develop novel therapeutic drugs with higher efficacy and safety. In this study, with a focused view on implementing an in silico drug design pipeline, a diverse set of glycosylated flavonoids were screened against the Mtb cytochrome-P450 enzyme 121 (CYP121), which is established as an approved drug target for the treatment of Mtb infection. A total of 148 glycosylated flavonoids were screened using structure-based virtual screening against the crystallized ligand, i.e., the L44 inhibitor, binding pocket in the Mtb CYP121 protein. Following this, only the top six compounds with the highest binding scores (kcal/mol) were considered for further intermolecular interaction and dynamic stability using 100 ns classical molecular dynamics simulation. These results suggested a considerable number of hydrogen and hydrophobic interactions and thermodynamic stability in comparison to the reference complex, i.e., the CYP121-L44 inhibitor. Furthermore, binding free energy via the MMGBSA method conducted on the last 10 ns interval of MD simulation trajectories revealed the substantial affinity of glycosylated compounds with Mtb CYP121 protein against reference complex. Notably, both the docked poses and residual energy decomposition via the MMGBSA method demonstrated the essential role of active residues in the interactions with glycosylated compounds by comparison with the reference complex. Collectively, this study demonstrates the viability of these screened glycosylated flavonoids as potential inhibitors of Mtb CYP121 for further experimental validation to develop a therapy for the treatment of drug-resistant Mtb strains.

## 1. Introduction

Tuberculosis, an infectious disease caused by *Mycobacterium tuberculosis* (Mtb), is liable for approximately 10 million new cases and 1.6 million deaths per year [1]. Tuberculosis-related conditions are further classified into two categories based on the symptoms; these are (1) latent tuberculosis infection (LTBI) and (2) TB illness. The global rise in the cases of tuberculosis and the evolution of prolonged-drug-resistant (MDR and XDR) *Mycobacterium tuberculosis* (Mtb) strains demand effective diagnostic and therapeutic tools. Recently, phenotypic screening has provided a new therapeutic tool to screen heavy compound libraries to detect the inhibitory molecule against Mtb [2,3,4]. In contrast, a structure-based therapeutic design has also been widely used due to the decipherment of the Mtb genome sequence in 1998, which helped to identify a plethora of additional potential targets [5,6,7,8,9]. This strategy starts with the identification of proteins that are essential for pathogens, and the structural co-ordinates of these drug targets are used to screen the library of chemical entities to estimate their molecular binding and efficacy. However, there are several additional challenges beyond molecular binding that affects the efficacy of a drug molecule. For example, the cell wall of *mycobacterium* is an overly complex biomolecular structure which creates a wax-like barrier with partially recognized permeability behavior. This demanding characteristic of *Mycobacterium tuberculosis* poses an additional challenge for novel drug-target inhibitors to reach their specific target and create the required physiological changes [10].

Mycocyclosin is a secondary metabolite that has a critical role in the survival of *Mycobacterium tuberculosis* [11]. Biochemical pathways studies suggested that mycocyclosin synthase (CYP121) is the enzyme responsible for producing mycocyclosin by catalyzing C-C bond formation between the carbons ortho to the phenolic hydroxyl of cyclo (L-tyr-L-tyr) (cYY). This enzyme is encoded by the *Cytochrome P450 121* gene. The involvement of CYP121 in essential pathways of Mtb marks it as a potential drug target to inhibit Mtb infections [12,13,14,15,16]. In recent years, several azole-containing compounds with known antifungal activity have been shown to exhibit promising antimycotic activity [12,15,17]. These azole-derived compounds showed high affinity to CYP121 with probable inhibitory action against the growth of Mtb. The MIC values for several azole derivatives, including econazole, clotrimazole, and miconazole, were reported to be 8, 11, and 8 g/mL, respectively, with a strong dissociation constant for binding with CYP121 [12,13]. In this list, the 4-iodopyrazole compound was reported as the most potent experimentally validated small-molecule inhibitor of the CYP121 protein [18]. Likewise, the (3S,6S)-3,6-bis (4-hydroxybenzyl) piperazine-2,5-dione compound is another strong inhibitor of the CYP121 protein (Kd of 10.5 µM) listed in the drug bank database [19,20]. Moreover, the mechanism of action of these inhibitors was deciphered as an interference with the substrate conversion for the CYP121 protein [21,22]. Furthermore, in silico approaches for rational drug design for CYP121 were demonstrated in several earlier studies. For instance, isoniazid hydrazones were assessed as potential mycobacterial CYP121 binders using molecular docking, and their cell penetration was also experimentally validated [23]. Computational methods are complemented with the experimental pipeline to examine the molecular interactions of inhibitory compounds with CYP121. In this context, Oxazolyl-Pyrimidines were predicted to have substantial interactions with CYP121 using docking protocols, while it was shown that anti-mycobacterial testing deciphered a highest MIC value of 3.12 µg/mL [24]. Similarly, the screening of 10,000 azole compounds against the CYP121 protein resulted in the 15 compounds with considerable binding energy and binding free energy [25]. Moreover, the critical residues, i.e., V83, F168, W182, D185, V228, and Q385, for the inhibition of CYP121 by the azole compounds were also elucidated [25]. Furthermore, ketoconazole was also analyzed for the binding and probable inhibitory potential against most of the *Mycobacterium* P450 enzymes [23]. With regard to azole derivatives, the anti-mycobacterium activity of 4-amino-5-(4-fluoro-3-phenoxy phenyl)-4H-1,2,4-triazole-3-thiol (1) and its Schiff bases was also experimentally tested and showed a considerable MIC value (5.5 g/mL) against a multi-drug-resistant (MDR) strain. Interestingly, this compound also showed considerable intermolecular interactions with the CYP121 protein [24]. Overall, earlier studies showed the high acceptability of in silico approaches to investigate the molecular interaction of existing and novel inhibitors of the CYP121 protein.

Considering the significant therapeutic index of natural compounds, they are being examined against several drug targets [26]. Flavonoids are the class of secondary plant metabolites with a diverse chemical composition known to exhibit relevant medicinal properties [27,28]. The broad spectrum of the therapeutic characteristic of flavonoids was examined and established, which includes antiviral, neuroprotective, anticancer, and anti-inflammatory properties [29,30]. In this study, a structure-based in silico drug design methodology was implemented to detect and investigate the potential binding of glycosylated flavonoid compounds against the CYP121 protein. The glycosylated flavonoid library was virtually screened against the binding pocket of the CYP121 protein to find the best binding poses of the compounds with the ability to alter the function of CYP121. The flexibility and dynamicity of the best-docked poses were also assessed using explicit solvent molecular dynamic (MD) simulation and evaluated for the MM/GBSA binding free energy. This resulted in the identification of six hit compounds, i.e., Catechin 3,7,-Di-*O*-Galate, Catechin 5,7,-Di-*O*-Gallatec, Catechin 5,4′-Di-*O*-Gallate, Epifisetinidol-(4beta->8)-Catechin, Catechin 3-O-Rutinoside, and Epicatechin (4b->6) Catechin, with high conformational and dynamic stability with the CYP121 protein.

## 2. Methodology

### 2.1. Protein Structure Preparation

The experimental crystal structure of the Mtb cytochrome-P450 enzyme 121 (CYP121) protein was sourced from the RCSB protein databank (PDB ID: 6TEV) at 1.70 Å resolution with the L44 inhibitor located at the entrance to the CYP121 active site [31]. Furthermore, the protein structure was processed by adding polar hydrogen atoms, which are responsible for critical H-bond interaction with the ligand, followed by the proper bond order assignment under default parameters using the dock prep program USCF Chimera-1.16 [32], as reported earlier [33].

### 2.2. Ligand Collection

A total of 148 glycosylated flavonoids, based on one of the Lipinski’s rules of five, also known as Pfizer’s rule of five—the molecular weight of compounds should be 180 to 480 g/mol to exhibit drug-likeness—were collected from the PubChem database [34].

### 2.3. Binding Site and Virtual Screening

To screen the selected glycosylated compounds, a docked grid of dimensions (x, y, and z = 17.7948, 18.4153, and 14.6274, respectively) centered at (x, y, and z = −10.7178, 14.1029, and 1.46746 Å) was generated around the residues interacting with the native ligand, i.e., the L44 inhibitor, in the prepared CYP121 protein structure (6TEV). In the crystal structure, the reference ligand bound in the hydrophobic binding pocket and showed both hydrophobic interactions as well as hydrogen bonding with the Mtb CYP121 protein [31]. Briefly, ligands in SDF files were prepared by conversion into the PDBQT format using the inbuilt OpenBabel tool [35] and energy minimized by the Universal Force Field [36]under the default parameters in the PyRx0.8 (Virtual Screening Tools), as reported earlier (cite it: https://doi.org/10.1016/j.molliq.2020.113322). Following this, the virtual screening of the prepared ligands was docked using the AutoDock Vina tool under default parameters, including 10 binding modes, an exhaustiveness of 8, and a maximum energy difference of 3 (kcal/mol) using the AutoDock Vina in PyRx tool [37,38].

### 2.4. Redocking and Molecular Contact Analysis

The redocking simulation between the Mtb CYP121 protein and the selected best conformation of glycosylated flavonoids was conducted using the Chimera-AutoDock Vina plugin setup to refine the interactions for the respective complexes. Briefly, the receptor and ligand libraries were prepared using the Dock prep tool in Chimera under default parameters, and molecular docking simulations were performed using the AutoDock Vina [38] plugin with the default setting at the native ligand binding pocket by adopting the docking grid size of 17.7948, 18.4153, and 14.6274 Å along all three (X, Y, and Z) axes, covering all the essential residues in the center at the −10.7178, 14.1029, and 1.46746 Å regions, to provide copious space for the ligand conformations. By docking, at least 10 conformers were generated and conformed with the lowest binding energy and RMSD, which were chosen for further analysis in free academic Maestro v12.3 (Schrödinger Release 2020-1: Maestro, Schrödinger, LLC, New York, NY, USA, 2020). Herein, non-covalent interactions, viz. hydrogen bonding, hydrophobic, π-π, π-cation, positive (basic or positive amino acids), negative (acidic or negative amino acids), polar (polar amino acids), glycine (non-polar interaction), and salt bridge interactions, were calculated at the cutoff radius of 4 Å under default conditions, and both 3D and 2D interaction images were generated. A similar docking methodology was also employed for the crystal structure ligand, i.e., with the L44 inhibitor [31], to validate the docking procedure and for comparative analysis with the selected antibiotics.

### 2.5. Explicit Molecular Dynamics Simulation

GROMACS 4.6.2 [39] with the CHARMM27 force field was used to run the molecular dynamic simulation for the selected docked complexes [40]. The protonation state of the protein was maintained at the physiological condition during the simulation. The CGenFF tool was used to build the topology and parameters for small molecules that were consistent with the CHARMM all-atom force field [41]. Maintaining the physiological settings, hydrogen atoms were added to the protein and centered in a solvated box positioned at 1.4 nm from the wall of the solvated cubical box. The protein–ligand solvated complex was energetically minimized over 5000 steps using the steepest deepest algorithm. The SHAKE algorithm was employed with a time step of 2 fs. Further, during the equilibration process, the system was subjected to a constant temperature (NVT) and pressure (NPT) ensemble for 100 ps and 1 ns, respectively. As per the standard protocol, ligand and protein molecules were constrained during the equilibrium phase, and only solvent was allowed for motion. Once the complex was equilibrated, a 100 ns all-atom simulation was carried out using the V-rescale temperature coupling method [42] connected with an external heat bath with a 0.1 ps time constant for the protein and ligand, while pressure coupling was deployed using the Parrinello-Rahman [43] method with a time constant of 2 ps; long-range electrostatics were handled using the PME (particle mesh Ewald) method [44,45]. Likewise, complexes without ligands, termed Apo-protein, and complexes with L44 inhibitors, marked as reference complexes, were also simulated under similar conditions. Finally, all the generated MD simulation trajectories were analyzed using the GROMACS utilities.

### 2.6. End-Point Binding Free Energy Calculation

Post simulation, the last 10 ns simulation trajectory was used to calculate the Molecular mechanics/generalized Born Surface area (MM/GBSA) binding free energies using gmx_MMPBSA v1.5.5 in an Anaconda environment [46]. This tool is an implementation of the AMBER MMPBSA program onto Gromacs compatible files. It uses the AMBER and CHARMM force fields [47] for parameterization. The binding free energy has multiple components that are essentially categorized into (a) molecular mechanics, (b) polar solvation energy, and (c) non-polar solvation energy. The illustration of binding free energy is shown in Equation (1).

(1) $$\Delta G_{\mathrm{Bind}}=\Delta G_{Complex}-\left(G_{\mathrm{Receptor}}-G_{\mathrm{Ligand}}\right)$$

where, ∆*G*_bind_ = ∆H − T∆S; ∆H = ∆*E*_MM_ + ∆*G*_sol_ + ∆*E*_VDWg_; ∆*E*_MM_ = ∆*E*_bonded_ + ∆*E*_non-bonded_; ∆*G*_sol_ = ∆*G*_polar_ + ∆*G*_non-polar_; $\Delta G_{\mathrm{Bind}}$: Change in Binding free energy; $G_{Complex}$: Free energy of the complex; $G_{\mathrm{Receptor}}$: Free energy for the receptor; $G_{\mathrm{Ligand}}$: Free energy for the ligand; ∆H_:_ Change in enthalpy; ∆S_:_ Change in entropy (neglected in this equation ≈ 0); ∆*E*_MM:_ Change in molecular mechanics; ∆*E*_bonded:_ Change in bonded energy (bond, angle, dihedral); ∆*E*_non-bonded:_ Change in non-bonded energy (electrostatic, van der Waal); ∆*G*_sol:_ Change in solvation energy; ∆*G*_pol:_ Change in Polar Solvation energy; ∆*G*_non-polar:_ Change in non-polar solvation energy.

## 3. Results and Discussion

### 3.1. Virtual Screening Analysis

In this study, a total of 148 glycosylated flavonoids were retrieved from the PubChem database, which are mentioned in Table S1, with ranges in molecular weights (110 to 615 g/mol), formal charges (−1 to 3), rotatable bonds (0 to 11), polar surface areas (40 to 280 2 Å^2^), hydrogen donors (0 to 12), hydrogen acceptors (2 to 16), and XlogP (−3.4 to 4.1). Additionally, 1-[[4-[4-(trifluoromethyl)phenyl]phenyl]methyl]imidazole, also known as N5W/L44 in the crystal structure of the protein data bank file, was used as a reference inhibitor. It has a molecular weight of 302.29 g/mol, XlogP of 4.1, three rotatable bonds, a polar surface area of 17.822 Å^2^, 0 hydrogen donors, and four hydrogen acceptors. Of note, the glycosylated compounds showed chemical properties within the range of the reference compound N5W/L44. Following this, the virtual screening was performed at the defined docking grid containing essential residues interacting with the reference inhibitor using the AutoDock Vina-PyRx tool setup [38]. Table S2 shows the PubChem IDs of all the selected flavonoid compounds and their respective biding affinities (kcal/mole) with the Mtb CYP121 protein. The analysis of the virtual screening revealed a binding affinity between −11.2 kcal/mol and −4 kcal/mol for the best poses of screened compounds in the Mtb CYP121 protein. (Table S2).

### 3.2. Re-Docking Top Hits and Molecular Interaction Analysis

To refine the binding interactions of the active residues in the selected binding pocket of the Mtb CYP121 protein, the top six compounds were collected from the virtual screening for redocking and intermolecular interaction analysis (Table 1). The analysis of the best poses from the redocking analysis showed docking scores between −10.7 and −9.8 kcal/mol. Herein, Catechin 3,7, -Di-*O*-Galate (10.7 kcal/mol) and Epicatechin (4b->6) Catechin (−9.8 kcal/mol) were noted for their highest and lowest binding scores with the Mtb CYP121 protein. Additionally, all the docked compounds have higher binding energy by comparison to the reference ligand (−9.1 kcal/mol), as shown in Table 1. Thus, only the top poses of the selected compounds with relatively highest binding scores were considered for the intermolecular interaction analysis using the free academic version of Maestro v12.3 suite.

**Figure 1:**
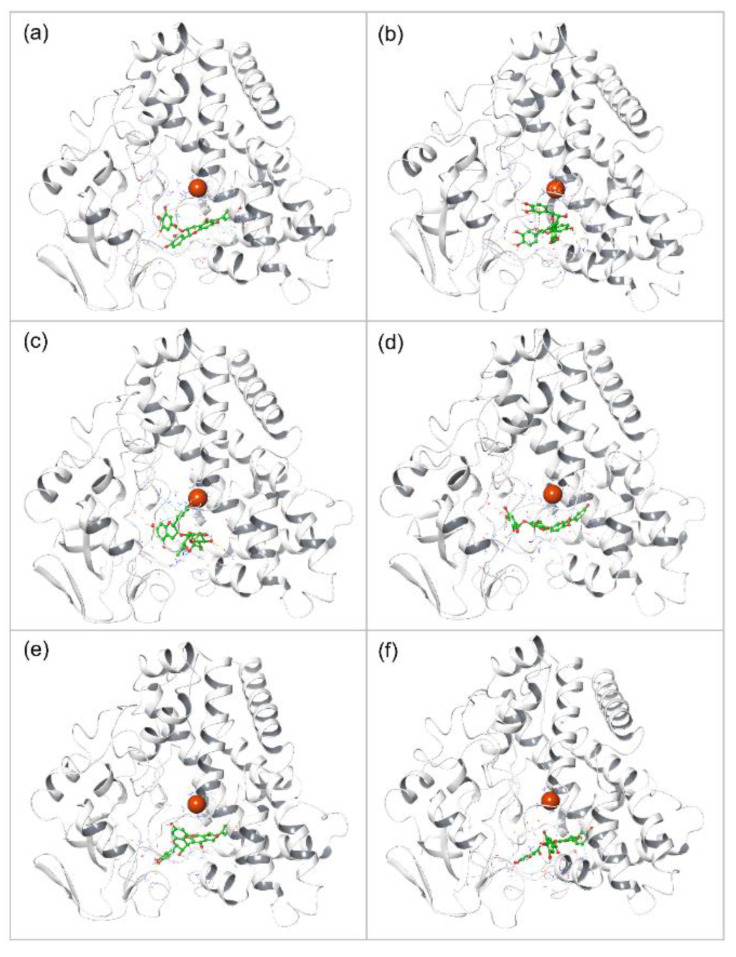
Three-dimensional interaction illustration of protein binding with ligands (**a**) Catechin 3,7,-Di-*O*-Galate, (**b**) Catechin 5,7,-Di-*O*-Gallatec, (**c**) Catechin 5,4′-Di-*O*-Gallate, (**d**) Epifisetinidol-(4beta->8)-Catechin, (**e**) Catechin 3-*O*-Rutinoside, (**f**) Epicatechin (4b->6) Catechin. (Light Gray->Protein, Green->Ligand, Red ball/blue residue->Heme co-factor).

As shown in Figure 1, Figures S1 and S2, all the selected top six glycosylated flavonoid compounds were observed to form hydrogen bonds with the active site residues of the Mtb CYP121 protein. Compound **1** (Catechin 3,7,-Di-*O*-Galate), with the best docking score of −10.7 kcal/mol, showed interactions with active site residues of Mtb CYP121 protein by forming hydrogen bonds with ASN74 (Figure 1 and Figure S1). Compound **2**, Catechin 5,7, -Di-*O*-Gallate, showed a −10.4 kcal/mol binding energy and formed stacking interaction with the ring of HIS343, as shown in (Figure 1 and Figure S1). This residue is an experimentally annotated binding site residue that interacts with the heme group [21]. Moreover, like compound **1**, this compound was also involved in the formation of an H-bond with ASN74 to achieve greater binding stability. Compound **3**, Catechin 5,4′-Di-*O*-Gallate, showed a −10.3 kcal/mol binding score and formed four H-bonds with the receptor molecule. These hydrogen bonds were formed with ASN74, ASN85, ALA233, and GLN385, as shown in Figure 1 and Figure S1.

The stacking interaction for this molecule was found with PHE280. It was experimentally noted that ASN85, which is listed in its H-bond interaction, belongs to a core substrate-binding site. Compound **4**, Epifisetinidol-(4beta->8)-Catechin, had a binding score of −9.8 kcal/mol and formed H-bonds with ASN74 and ASP282 as well as a stacking interaction with PHE168, as shown in Figure 1 and Figure S1. The binding energy of compound **5**, catechin 3-*O*-Rutinoside, was −9.8 kcal/mol. This molecule formed a single H-bond with THR65 residues, as shown in Figure 1 and Figure S1. Compound **6**, Epicatechin (4b->6) Catechin, had a −9.8 kcal/mol binding energy with three H-bonds. These H-bonds were formed by THR77, MET86, and GLN385, as shown in Figure 1 and Figure S1. It also had a stacking interaction with TRP182. Here, THR77 is known as the critical substrate-binding residue [22,48] while TRP182 is involved in stacking interaction with the natural substrate. ASN85-MET86 is also considered a critical binding region for the substrate, and MET85 forms a direct hydrogen bond with Compound **6**. Furthermore. the redocking of the reference inhibitor L44 showed a binding score of −9.1 kcal/mol and showed interactions by H-bond (ARG386) and pi-pi stacking (PHE168). Additionally, all the docked complexes were noted for hydrophobic, polar, negative, and positive residual interactions within 4 Å around the ligand with the Mtb CYP121 protein (Figure S2). Interestingly, in the crystal structure of Mtb CYP121 [31], the inhibitor also showed proximity to the ASN74 residue. Additionally, this compound has stacking (ring) interaction with TRP182. CYP121 receptor enzymatic activity involves the formation of a C-C bond with its substrate, where TRP182 interacts with the substrate via stacking. Inhibitor interaction with this residue can play a critical role in affecting the enzymatic activity of CYP121. Moreover, similar interactions were found in the docked poses of the glycosylated flavonoids and the reference L44 inhibitor, which suggests the considerable affinity of the screened compounds with the Mtb CYP121 protein.

### 3.3. Molecular Dynamics Analysis

Deciphering the overall dynamics of protein–ligand interaction is significantly aided by the application of MD simulations. The interaction behavior of each ligand with CYP121 was determined using a 100 ns explicit solvent MD simulation. Before the 100 ns production phase, the complete system was equilibrated in a solvated box to settle the water molecules and ions.

#### 3.3.1. RMSD Analysis

**Figure 2:**
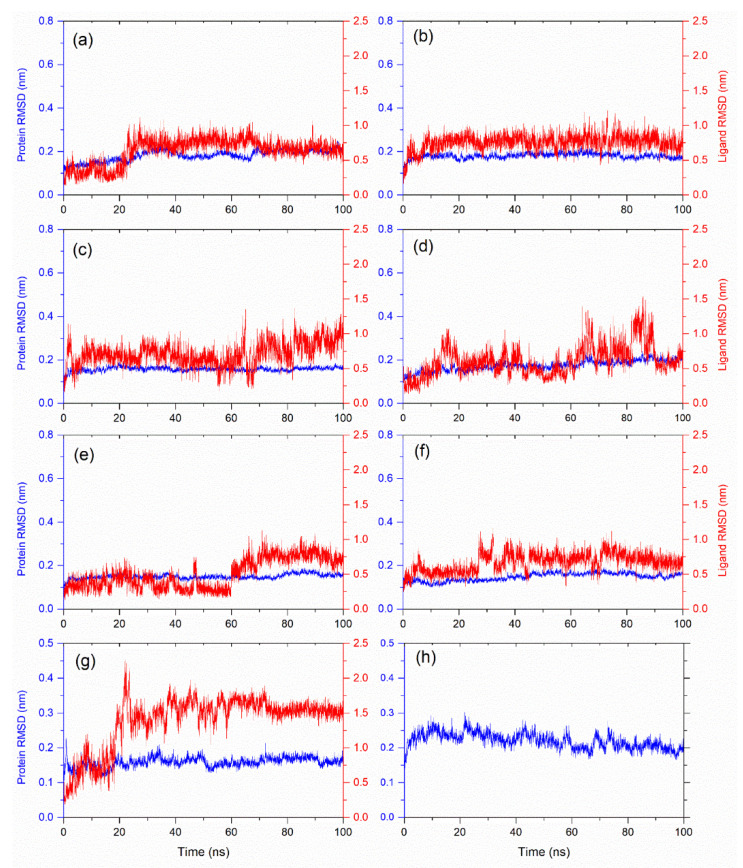
Root mean square deviation (RMSD) of protein and ligand for the docked poses obtained from 100 ns MD simulation for (**a**) Catechin 3,7,-Di-*O*-Galate, (**b**) Catechin 5,7,-Di-*O*-Gallatec, (**c**) Catechin 5,4′-Di-*O*-Gallate, (**d**) Epifisetinidol-(4beta->8)-Catechin, (**e**) Catechin 3-*O*-Rutinoside, (**f**) Epicatechin (4b->6) Catechin, (**g**) L44 inhibitor and (**h**) apo-protein. Cα atoms of protein were used for RMSD calculation (blue), and ligand RMSDs (red) were calculated for heavy atoms by fitting the protein-ligand complex.

The root mean square deviation (RMSD) across the trajectory is the measurement that determines the deviation of the molecule from its initial conformation. In this case, the RMSD was computed for the complete trajectory for both the protein and the ligand independently to provide the conformational variation within the system. In a molecular comparison, an RMSD less than 0.3 nm (3 Å) is considered acceptable, which signifies no or minimum change [49]. Figure 2 displays the root mean square deviation (RMSD) for the protein and docked ligands. Interestingly, all the docked complexes showed substantial stability in the protein structure in comparison to the reference complex and apo-protein. Likewise, the analysis of the RMSD values for the docked glycosylated flavonoids (<1 nm) showed considerable values by comparison to the L44 inhibitor (>1 nm). For instance, it was observed that compound **2** showed the most stable pattern and attained it at the early stage of simulation (~10 ns). Furthermore, compound **1** demonstrated a consistent pattern at the start of the simulation with an RMSD of 0.3 nm and showed this behavior until 20 ns. Later, it took a major spike and reached 0.7 nm and stabilized there for the rest of the 80 ns of the simulation. After compounds **2** and **1**, compound **6** showed the minimum structural deviation; it started with an RMSD of 0.3 nm and remained stable within the range of 0.6 nm until 30 ns of the simulation had passed. Later, the RMSD increased but remained relatively constant throughout the simulation at approximately 0.7 nm. Compound **5** showed peculiar behavior in which the deviation could be categorized into two phases. It showed a stable pattern with an RMSD of 0.3 nm in the first phase of 0–60 ns, while in the second phase, it fluctuated to approximately 0.7 nm. During the simulation, compounds **3** and **4** showed the most variation, with compound **4** being the most unstable among the two because it often reached an RMSD value of 1.5 nm. These observations were in accordance with the docking results, where higher docking scores were noted for the selected glycosylated flavonoids in comparison to the reference inhibitor (Table 1). Hence, based on the molecular dynamics’ simulation analysis, all the screened flavonoids were suggested to be potential inhibitors of the Mtb CYP121. Based on the ligand RMSD analysis, these flavonoids can be arranged in the order of their stability with the bacterial protein, viz. Catechin 3,7, -Di-*O*-Galate, Catechin 5,7,-Di-*O*-Gallatec, Epicatechin (4b->6) Catechin, Catechin 5,4′-Di-*O*-Gallate, Epifisetinidol-(4beta->8)-Catechin, and Catechin 3-*O*-Rutinoside. Additionally, to study the docked ligands’ stability with respect to time, MD simulation trajectories were converted to form the simulation movies and provided Supporting Information.

#### 3.3.2. RMSF Analysis

**Figure 3:**
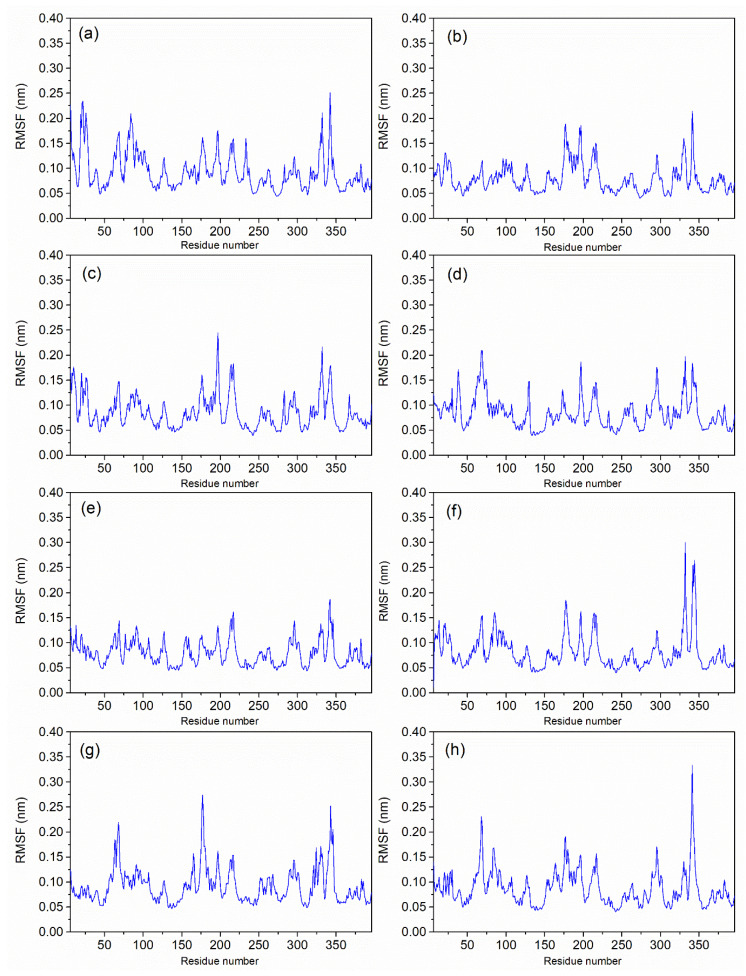
RMSF plots of protein with all ligands (**a**) Catechin 3,7,-Di-*O*-Galate, (**b**) Catechin 5,7,-Di-*O*-Gallatec, (**c**) Catechin 5,4′-Di-*O*-Gallate, (**d**) Epifisetinidol-(4beta->8)-Catechin, (**e**) Catechin 3-*O*-Rutinoside, (**f**) Epicatechin (4b->6) Catechin, (**g**) reference L44 inhibitor, and (**h**) apo-protein.

As shown in Figure 3, the root mean square fluctuation (RMSF) was computed for both the protein and each docked protein fit ligand from the 100 ns MD simulation trajectories. RMSF is a numerical measurement that is remarkably similar to RMSD, but it calculates the conformational jump for individual residues. The RMSF per residue is often plotted against the number of residues, and it may indicate the amino acids in a protein that contribute the most to its molecular motion. In this investigation, the RMSF for all residues stayed under 0.3 nm for the course of the complete simulation.

Binding pocket residues were closely examined for RMSF to estimate its effect on ligand binding. In the compound **1** complex (Figure 3a), only HIS343 out of 19 binding pocket residues (62, 86, 146, 230, 233, 234, 237, 238, 241, 280, 284, 286, 337, 338, 339, 343, 345, 346, and 347) showed a relatively higher RMSF (0.2 nm). There was no evidence of any significant RMSF in any of the binding site residues in the complexes of compounds **2** and **4**, as shown in Figure 3b,d. Additionally, the compound **3** complex showed HIS343 from the binding site in the top 10 RMSF values with a score of 0.18 nm (Figure 3c). Figure 3e shows the RMSF for the protein complexed with compound **5** and it has PRO346, a binding site residue, listed in the top 10 maximum RMSFs. There were three residues from the binding site which indicated an RMSF jump (shown in Figure 3f) for compound **6**; these residues were CYS345, HIS343, and PRO346. Overall, the RMSFs of all critical binding site residues were under 0.3 nm, which suggested the marginal fluctuation of the binding site of the protein during the interaction with all six hit compounds. All the docked complexes with glycosylated flavonoids showed considerable RMSF values in comparison to the reference complex (Figure 3g) and apo-protein (Figure 3h). These data suggested that docked flavonoids have contributed to the overall stability of the protein structure as per the 100 ns MD simulation interval.

**Figure 4:**
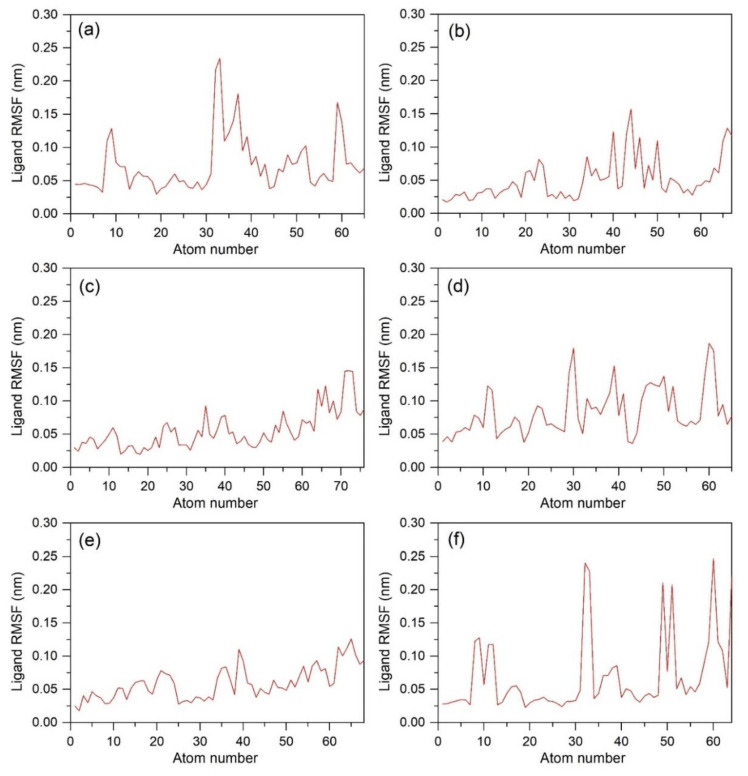
RMSF (root mean square fluctuation) for all atoms for each compound (**a**) Catechin 3,7,-Di-*O*-Galate, (**b**) Catechin 5,7,-Di-*O*-Gallatec, (**c**) Catechin 5,4′-Di-*O*-Gallate, (**d**) Epifisetinidol-(4beta->8)-Catechin, (**e**) Catechin 3-*O*-Rutinoside, (**f**) Epicatechin (4b->6) Catechin.

Figure 4 shows the RMSF of each atom of the six ligands complexed with a protein receptor. Even though all the values were under the threshold of 0.3 nm, there were still certain peaks observed, as shown in Figure 4. The compounds **2**, **3**, **4**, and **5**, shown in Figure 4b–d,e, did not show any RMSF over 0.2 nm. However, in compounds **1** and **6**, shown in Figure 4a,f, there were certain atoms detected that showed RMSFs greater than 0.2 nm. In compound **1**, these atoms were O32 and C33, while for compound **6**, these atoms were H60, O32, C33, H64, H49, and H51. Overall, none of the atoms in any hit compound showed any significant fluctuations by comparison to the reference L44 inhibitor (Figure S3) and were thus marked for substantial stability with the bacterial protein during the 100 ns MD simulation interval.

#### 3.3.3. Binding Free Energy and Residue Decomposition Analysis

**Figure 5:**
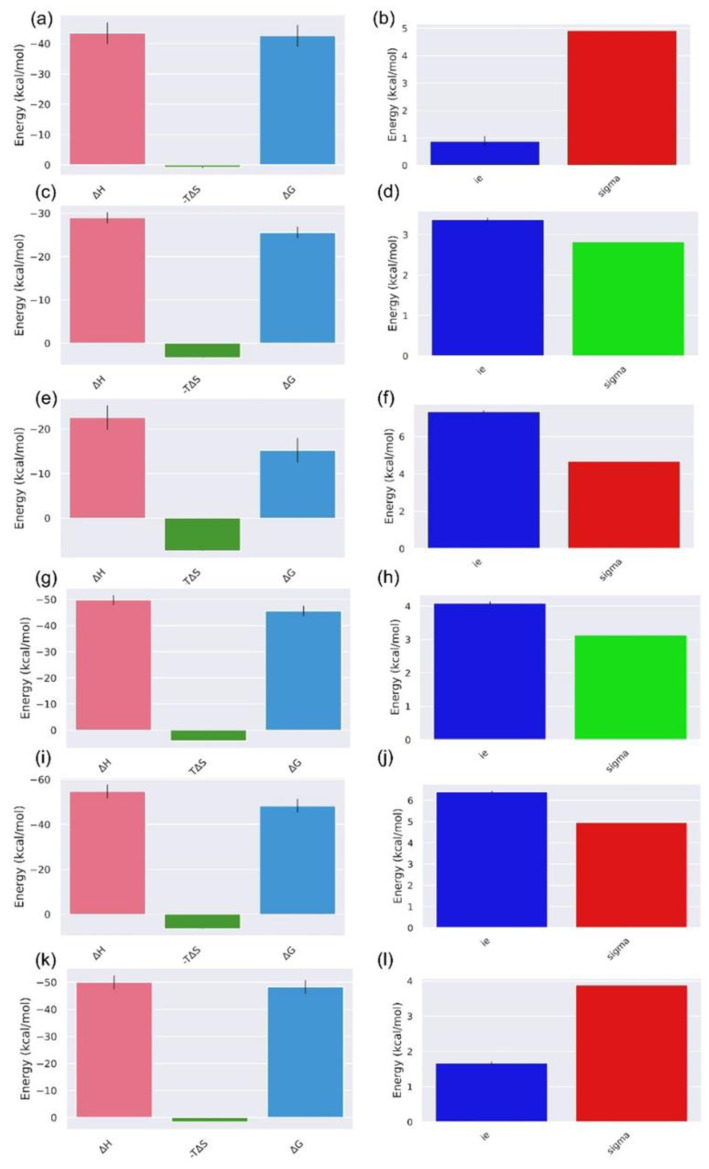
Enthalpy (ΔH), Entropy (TΔS), Binding free energy (ΔG), Interaction Entropy (IE), and Sigma (standard deviation of the interaction energy) values for compounds (**a**,**b**) Catechin 3,7,-Di-*O*-Galate, (**c**,**d**) Catechin 5,7,-Di-*O*-Gallate, (**e**,**f**) Catechin 5,4′-Di-*O*-Gallate, (**g**,**h**) Epifisetinidol-(4beta->8)-Catechin, (**i**,**j**) Catechin 3-*O*-Rutinoside, (**k**,**l**) Epicatechin (4b->6) Catechin complex with CYP121 protein.

Figure 5 depicts the total binding free energies and their various energy components, representing enthalpy (ΔH) and entropy (TΔS). ΔH shows the energy in reference to heat evolved or absorbed in a reaction carried out at constant pressure, while TΔS represents the degree of disorder or randomness in the system.

**Figure 6:**
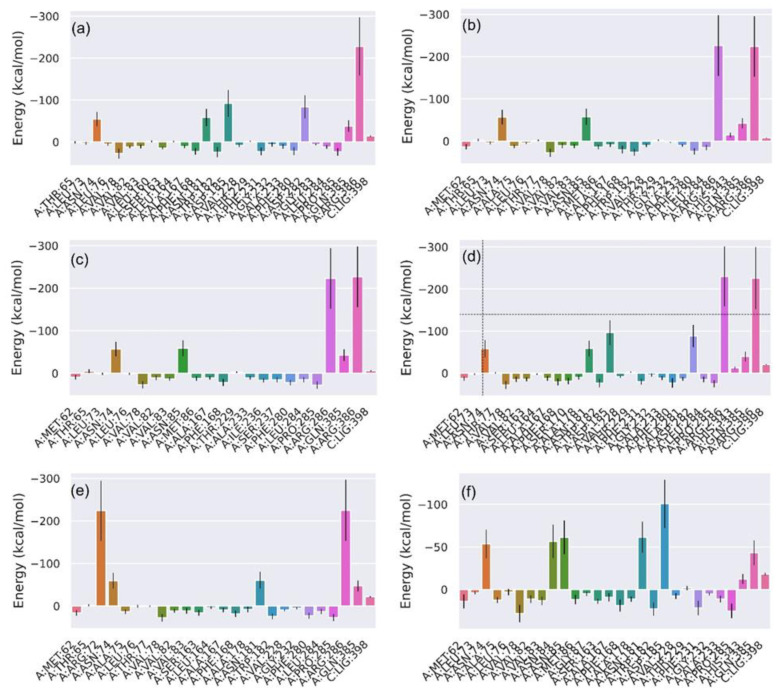
Free energy residue decomposition for binding site residues of CYP121 complexed with (**a**) Catechin 3,7,-Di-*O*-Galate, (**b**) Catechin 5,7,-Di-*O*-Gallate, (**c**) Catechin 5,4′-Di-*O*-Gallate, (**d**) Epifisetinidol-(4beta->8)-Catechin, (**e**) Catechin 3-*O*-Rutinoside, (**f**) Epicatechin (4b->6) Catechin with protein complex.

Considering the enthalpies of all six compound complexes, it was observed that compounds **1**, **4**, **5**, and **6** almost showed a similar trend with scores of ΔH −42.61, −49.73, 57.64, and −49.97 kcal/mol, respectively (as shown in Figure 5, where the minimum score is shown for compound **5**, while compounds **2** and **3** show ΔH −28.94 and −22.57 kcal/mol (as shown in Figure 6). Furthermore, compound **1** had the lowest interaction entropy component of −0.88 kcal/mol, followed by compounds **6**, **2**, **4**, **5**, and **3**. The interaction entropy average is shown by the first bar in Figure 5. Their standard deviation (σ) of interaction entropy is also shown in these figures, represented by the second bar. It is known that if σ (interaction entropy) > 3.6 kcal/mol, then the entropy term is not reliable [50]. In all the complexes except for compound **2**, the σ (in-teraction entropy) > 3.6 kcal/mol. This suggests the approximation of entropy was not reliable in the calculation, but it can be considered for the relative ranking. Interestingly, all the docked complexes with glycosylated flavonoids showed higher binding affinity in comparison to the reference inhibitor (−22.97 kcal/mol), as shown in Figure S4. Collectively, glycosylated flavonoids were noted to have higher binding affinity in the binding pocket of the Mtb CYP121 protein.

Eventually, ∆G (change in free energy) was calculated, as shown in Figure 5. It showed a similar pattern to enthalpy: compounds **1**, **4**, **5**, and **6** showed ∆G −43.49, −45.69, −54.64, and −48.29 kcal/mol, with compound **5** having the lowest ∆G; however, compounds **2** and **3** showed ∆G values that were marginally lower than −20 kcal/mol and −15 kcal/mol, respectively. The contribution of binding site residues in ligand binding was calculated by their ΔG decomposition. The individual energies of all these residues are shown in Figure 6. As illustrated in Figure 6a, for the compound **1** complex, a total of nine residues were observed with negative ∆G scores. Moreover, ARG386, ASP185, and ASP282 showed the highest negative energies, with ΔG values of −249.90, −101.87, and −92.49 kcal/mol, respectively. The protein complexed with compound **2** also showed nine residues with negative energies; the top three of them were ARG286, ARG386, and ASN85 with ΔG scores of −249.34 kcal/mol, −246.13 kcal/mol, and −63.55 kcal/mol, respectively. In addition, it possessed three experimental substrate binding residues, ARG286, ASN85, and THR77, with negative ΔG contributions. In the compound **3** complex, the residues ARG286 (−249.16 kcal/mol), ARG386 (−245.77 kcal/mol), and ASN85 (−64.84 kcal/mol) were the top three ΔG-contributing residues, while ARG286, ASN85, and THR77 were from the experimental substrate binding list with negative ΔG scores. Compound **4** had ten residues with negative energies. Herein, the three most negatively ΔG-contributing residues were ARG286, ARG386, and ASP185 with their ΔG scores of −252.71, −248.53, and −105.33 kcal/mol, respectively. Three experimentally known substrate binding residues, ARG286, HIS343, and THR77, also had negative ΔG scores. Only five residues in compound **5** had negative ΔG energies; the top three residues were ARG286, ARG72, and ASN181, with scores of −247.38, −246.42, and −66.79 kcal/mol, respectively. ARG286 is the only experimentally known substrate binding residue in this list for the compound **5** complex. The compound **6** complex had ten residues with negative ΔG scores; the top three were ASP185, ASN85, and ASN181 with values of −109.78, −67.55, and −67.41 kcal/mol, respectively. It showed two experimentally known substrate binding residues, ASN85 and THR77, with ΔG values of −67.55 and −0.39 kcal/mol, respectively. Additionally, residual decomposition analysis for the reference L44 inhibitor showed the least residual interactions in comparison to the docked flavonoids (Figure S5), with suggested the substantial residual interactions with the glycosylated compounds with the active residues of the Mtb CYP121 protein.

## 4. Conclusions

In this study, a viral screening approach was used to screen glycosylated flavonoids against the CYP121 protein target of *Mycobacterium tuberculosis*. The study was based on molecular docking and molecular dynamic simulations. A co-crystallized ligand from the PDB structure was used as a reference for comparison. The top six hits from docking based on their binding energies were simulated for 100 ns under an explicit solvent condition. Compound **5**, Catechin 3-*O*-Rutinoside, showed the lowest ΔG binding energy calculated over the simulation trajectory. The results suggest that these hit compounds bind at the binding site with higher stability by forming multiple H-bonds. Experimentally known substrate binding residues are also involved in H-bonding, which indicate the probable inhibitory mechanisms of these compounds. The computational findings presented in this study need experimental validation to detect the biological activity of the hit compounds.

## Figures and Tables

**Table 1:** Redocking scores of top 10 compounds produced after virtual screening.

Compounds	PubChem IDs	Names	Binding Affinity	Docking Score
**1**	14583619	Catechin 3,7,-Di-*O*-Galate	−11.2	−10.7
**2**	15689621	Catechin 5,7,-Di-*O*-Gallate	−10.5	−10.4
**3**	15689620	Catechin 5,4′-Di-*O*-Gallate	−10.7	−10.3
**4**	14332863	Epifisetinidol-(4beta->8)-Catechin	−10.9	−9.8
**5**	44257079	Catechin 3-*O*-Rutinoside	−10.9	−9.8
**6**	131752345	Epicatechin(4b->6)Catechin	−10.6	−9.8
Reference	L44	1-[[4-[4-(trifluoromethyl)phenyl]phenyl]methyl]imidazole	--	−9.1

## Data Availability

The datasets used and/or analyzed during the current study are available from the corresponding author on reasonable request.

## Supplementary Materials

The following supporting information can be downloaded at: https://www.mdpi.com/article/10.3390/biom12101356/s1, Figure S1: Two-dimensional interaction plot between protein and top 6 hit compounds; Figure S2: Interaction plot between protein and reference inhibitor; Figure S3: RMSFs (root mean square fluctuations) for all atoms for reference inhibitor L44; Figure S4: Binding free energy and residue decomposition score of the reference inhibitor L44 with the Mtb CYP121 protein; Figure S5: Free energy residue decomposition for binding site residues of the reference inhibitor L44 with the Mtb CYP121 protein; Table S1: Chemical properties of glycosylated flavonoid small-molecule library; Table S2: Binding score of glycosylated small molecules library with CYP121.
