# Supplementary material for: Glycosylated Flavonoid Compounds as Potent CYP121 Inhibitors of Mycobacterium tuberculosis
Source: Biomolecules. 2022 Sep 23;12(10):1356. doi: 10.3390/biom12101356 (PMC9599785; doi:10.3390/biom12101356)
Supplement: Supplementary file 1 [file biomolecules-12-01356-s001.zip › biomolecules-1860340-supplementary-conversion.pdf]

Supplementary

Table S1. Chemical properties of glycosylated flavonoids small molecules library.

cid	mw	Polararea	xlogp	Hbonddonor	Hbondacc	Rotbonds
289	110.11	40.5	0.9	2	2	0
1203	290.27	110	0.4	5	6	1
9064	290.27	110	0.4	5	6	1
65064	458.4	197	1.2	8	11	4
72276	290.27	110	0.4	5	6	1
73160	290.27	110	0.4	5	6	1
73533	422.4	169	-1.4	7	10	3
107905	442.4	177	1.5	7	10	4
124025	576.5	210	2.4	9	12	2
130556	578.5	221	2.4	10	12	3
131795	614.5	269	-3.4	11	16	7
146798	578.5	221	2.4	10	12	3
147299	578.5	221	2.4	10	12	3
155660	388.5	99.4	3.8	4	6	8
156680	562.5	201	2.7	9	11	3
176920	320.29	120	0.3	5	7	2
182232	290.27	110	0.4	5	6	1
182659	346.4	66.4	1.7	1	6	5
367141	442.4	177	1.5	7	10	4
440833	306.27	131	-0.8	6	7	1
442684	546.5	180	3.1	8	10	3
471393	442.4	177	1.1	7	10	4
474540	578.5	221	2.4	10	12	3
474541	578.5	221	2.4	10	12	3
474542	592.5	230	2	10	13	2
3009240	416.5	116	3.9	4	7	9
3363314	500.4	141	2.4	0	11	11
5276454	442.4	177	1.5	7	10	4
5315742	500.4	141	2.4	0	11	11
6324898	452.4	190	-1.4	8	11	4
6419835	442.4	177	1.5	7	10	4
9872976	576.5	210	2.4	9	12	2
9959902	578.5	221	2.4	10	12	3
9995184	304.29	99.4	0.7	4	6	2
10099701	578.5	221	2.5	10	12	3
10343836	452.4	157	2.2	6	9	2
10348185	578.5	221	1.5	10	12	3
10415767	576.5	210	2.4	9	12	2
10789789	452.4	190	-1.4	8	11	4
10840629	562.5	201	2.7	9	11	3
11067541	332.3	77.4	1.3	2	6	4
11187672	365.4	162	-0.4	6	8	4
11250133	578.5	221	2.4	10	12	3
11272988	562.5	190	2.1	8	11	4
11699925	594.5	241	2	11	13	3
11731408	562.5	201	2.7	9	11	3

12314425	422.4	169	-1.2	7	10	3
13279083	490.4	88.4	4.1	3	8	3
13279096	302.28	88.4	0.9	3	6	1
13831061	594.5	241	2	11	13	3
13831063	594.5	241	2	11	13	3
13990878	576.5	210	1.3	9	12	2
13990892	578.5	221	2.4	10	12	3
14015946	562.5	201	2.7	9	11	3
14104302	452.4	190	-1.2	8	11	4
14332862	562.5	201	2.7	9	11	3
14332863	562.5	201	2.7	9	11	3
14332899	304.29	99.4	0.7	4	6	2
14583619	594.5	244	2.2	9	14	7
14586216	560.5	190	2.7	8	11	2
15689618	442.4	177	1.1	7	10	4
15689619	594.5	244	1.8	9	14	7
15689620	594.5	244	1.8	9	14	7
15689621	594.5	244	1.8	9	14	7
16095186	452.4	201	-1	9	11	3
16095188	452.4	201	-1	9	11	3
16095190	452.4	201	-1	9	11	3
17860344	365.4	162	-0.4	6	8	4
21589943	582.6	196	1.9	7	12	8
21589944	582.6	196	2.5	7	12	8
21626704	436.4	169	-0.7	7	10	3
21626705	452.4	190	-1.4	8	11	4
21626713	452.4	201	-1	9	11	3
21633047	318.32	88.4	1	3	6	3
21633048	332.3	77.4	1.3	2	6	4
21676357	428.4	154	0.9	5	9	4
21676366	422.4	169	-1.1	7	10	4
24823045	578.5	221	2.4	10	12	3
44257072	422.4	169	-1.4	7	10	3
44257076	452.4	190	-1.2	8	11	4
44257078	436.4	169	-0.7	7	10	3
44257079	598.5	248	-2.9	10	15	6
44257080	422.4	169	-1.1	7	10	4
44257081	452.4	190	-1.4	8	11	4
44257085	452.4	190	-1.4	8	11	4
44257086	452.4	190	-1.4	8	11	4
44257087	436.4	169	-0.9	7	10	3
44257088	452.4	190	-1.4	8	11	4
44257089	452.4	190	-1.4	8	11	4
44257090	614.5	269	-3.2	11	16	7
44257091	614.5	269	-3.2	11	16	7
44257092	614.5	269	-3.2	11	16	7
44257093	614.5	269	-3.2	11	16	7
44257105	442.4	177	1.1	7	10	4
44257106	442.4	177	1.1	7	10	4
44257107	594.5	244	1.8	9	14	7
44257108	594.5	244	1.8	9	14	7

44257109	430.4	157	1.3	6	9	4
44257111	466.4	179	-1.1	7	11	5
44257125	304.29	99.4	0.7	4	6	2
44257153	468.4	210	-1.3	9	12	4
44257154	468.4	210	-1.3	9	12	4
44257155	452.4	190	-0.8	8	11	3
44257156	466.4	179	-0.5	7	11	4
44460468	576.5	210	2.4	9	12	2
51042196	578.5	221	2.4	10	12	3
51136539	422.4	169	-0.8	7	10	4
53349182	592.5	230	2	10	13	2
54020287	436.4	169	-0.7	7	10	3
70697937	578.5	221	2.4	10	12	3
71260071	466.4	207	-1.2	8	12	4
71307294	422.4	169	-1.4	7	10	3
71310843	293.25	110	0.4	5	6	1
71579294	466.4	207	-1.2	8	12	4
72193636	594.5	241	2	11	13	3
72193637	594.5	241	2	11	13	3
72193638	594.5	241	2	11	13	3
72193645	594.5	241	2	11	13	3
73346329	560.5	190	2.7	8	11	2
100947852	452.4	190	-1.4	8	11	4
100947853	614.5	269	-3.2	11	16	7
101449730	614.5	280	-3.2	12	16	6
101488387	576.5	210	2.4	9	12	2
101607233	396.5	126	3.7	5	6	3
101616633	370.3	162	-0.2	5	9	3
101656434	452.4	190	-1.4	8	11	4
101683293	562.5	201	2.7	9	11	3
101938452	562.5	201	3.3	9	11	3
101938453	562.5	201	2.7	9	11	3
102170448	422.4	169	-1.4	7	10	3
102188253	578.5	218	0.6	9	12	2
102232874	370.3	162	-0.2	5	9	3
102263677	452.4	201	-1	9	11	3
102263679	452.4	201	-1	9	11	3
102263681	436.4	180	-1.6	8	10	2
102263683	436.4	180	-0.5	8	10	2
102367837	560.5	190	2.7	8	11	2
102400061	578.5	221	2.4	10	12	3
102460989	452.4	190	-1.2	8	11	4
102460990	452.4	190	-1.2	8	11	4
102460991	614.5	269	-3.4	11	16	7
117908436	370.3	162	-0.2	5	9	3
124203170	445.3	177	1.5	7	10	4
131752345	578.5	221	1.6	10	12	3
131752346	578.5	221	1.6	10	12	3
131871365	293.25	110	0.4	5	6	1
133561693	436.4	169	-0.7	7	10	3
146026647	370.3	162	0	5	9	3

Table S2. Binding score of glycosylated small molecules library with CYP121.

Ligands	Binding Affinity	rmsd/ub	rmsd/lb
14583619	−11.2	0	0
14332863	−10.9	0	0
44257079	−10.9	0	0
10343836	−10.8	0	0
130556	−10.8	0	0
15689620	−10.7	0	0
21589944	−10.7	0	0
131752345	−10.6	0	0
15689621	−10.5	0	0
13279083	−10.5	0	0
13990878	−10.4	0	0
24823045	−10.4	0	0
44257107	−10.4	0	0
101938453	-10.2	0	0
147299	-10.2	0	0
367141	-10.2	0	0
107905	-10.2	0	0
11731408	-10.1	0	0
21589943	-10.1	0	0
102188253	-10.1	0	0
101938452	-10.1	0	0
15689619	-10	0	0
9959902	-10	0	0
101683293	−10	0	0
11699925	−10	0	0
44257105	−10	0	0
133561693	−10	0	0
44257072	−9.9	0	0
44257156	−9.9	0	0
21676357	−9.9	0	0
146798	−9.9	0	0
6419835	−9.8	0	0
10348185	−9.8	0	0
13831061	−9.8	0	0
44257088	−9.8	0	0
124203170	−9.8	0	0
11272988	−9.8	0	0
65064	−9.8	0	0
5276454	−9.7	0	0
70697937	−9.7	0	0
44257155	−9.7	0	0
101607233	−9.7	0	0
442684	−9.7	0	0
471393	−9.6	0	0
21676366	−9.6	0	0
44257078	−9.6	0	0
72193637	−9.6	0	0
44257080	−9.6	0	0
54020287	−9.6	0	0

131795	−9.6	0	0
102460991	−9.5	0	0
44460468	−9.5	0	0
71579294	−9.5	0	0
44257076	−9.5	0	0
14015946	−9.5	0	0
21626704	−9.4	0	0
13831063	−9.4	0	0
44257154	−9.4	0	0
21626705	−9.4	0	0
44257109	−9.4	0	0
15689618	−9.3	0	0
44257087	−9.3	0	0
44257086	−9.3	0	0
102460990	−9.3	0	0
44257093	−9.3	0	0
124025	−9.3	0	0
9872976	−9.3	0	0
5315742	−9.2	0	0
102263679	−9.2	0	0
12314425	−9.2	0	0
102263683	−9.2	0	0
117908436	−9.2	0	0
14104302	−9.2	0	0
72193645	−9.2	0	0
474540	−9.2	0	0
102232874	−9.2	0	0
16095186	−9.2	0	0
73533	−9.1	0	0
101616633	−9.1	0	0
102460989	−9.1	0	0
3363314	−9.1	0	0
14586216	−9.1	0	0
101656434	−9	0	0
474542	−9	0	0
14332862	−8.9	0	0
44257111	−8.9	0	0
51042196	−8.9	0	0
71307294	−8.9	0	0
16095190	−8.9	0	0
10789789	−8.9	0	0
44257085	−8.8	0	0
21626713	−8.8	0	0
102400061	−8.8	0	0
102170448	−8.8	0	0
71260071	−8.7	0	0
44257153	−8.7	0	0
102263681	−8.7	0	0
11250133	−8.7	0	0
474541	−8.7	0	0
10099701	−8.6	0	0

131752346	−8.6	0	0
51136539	−8.6	0	0
6324898	−8.6	0	0
10840629	−8.6	0	0
101488387	−8.6	0	0
156680	−8.6	0	0
44257081	−8.5	0	0
155660	−8.5	0	0
100947852	−8.5	0	0
13279096	−8.5	0	0
100947853	−8.5	0	0
176920	−8.5	0	0
13990892	−8.5	0	0
72276	−8.4	0	0
44257089	−8.4	0	0
1203	−8.4	0	0
3009240	−8.4	0	0
11187672	−8.4	0	0
146026647	−8.3	0	0
21633048	−8.3	0	0
44257106	−8.3	0	0
182232	−8.3	0	0
73160	−8.2	0	0
17860344	−8.2	0	0
71310843	−8.2	0	0
102263677	−8.2	0	0
14332899	−8.2	0	0
44257091	−8.1	0	0
10415767	−8.1	0	0
16095188	−8.1	0	0
9064	−8	0	0
21633047	−8	0	0
131871365	−8	0	0
440833	−8	0	0
182659	−7.9	0	0
44257125	−7.9	0	0
73346329	−7.9	0	0
9995184	−7.9	0	0
72193638	−7.8	0	0
44257090	−7.8	0	0
11067541	−7.8	0	0
44257092	−7.6	0	0
53349182	−7.6	0	0
72193636	−7.5	0	0
101449730	−7.4	0	0
102367837	−5.3	0	0
289	−5.1	0	0
44257108	−4	0	0

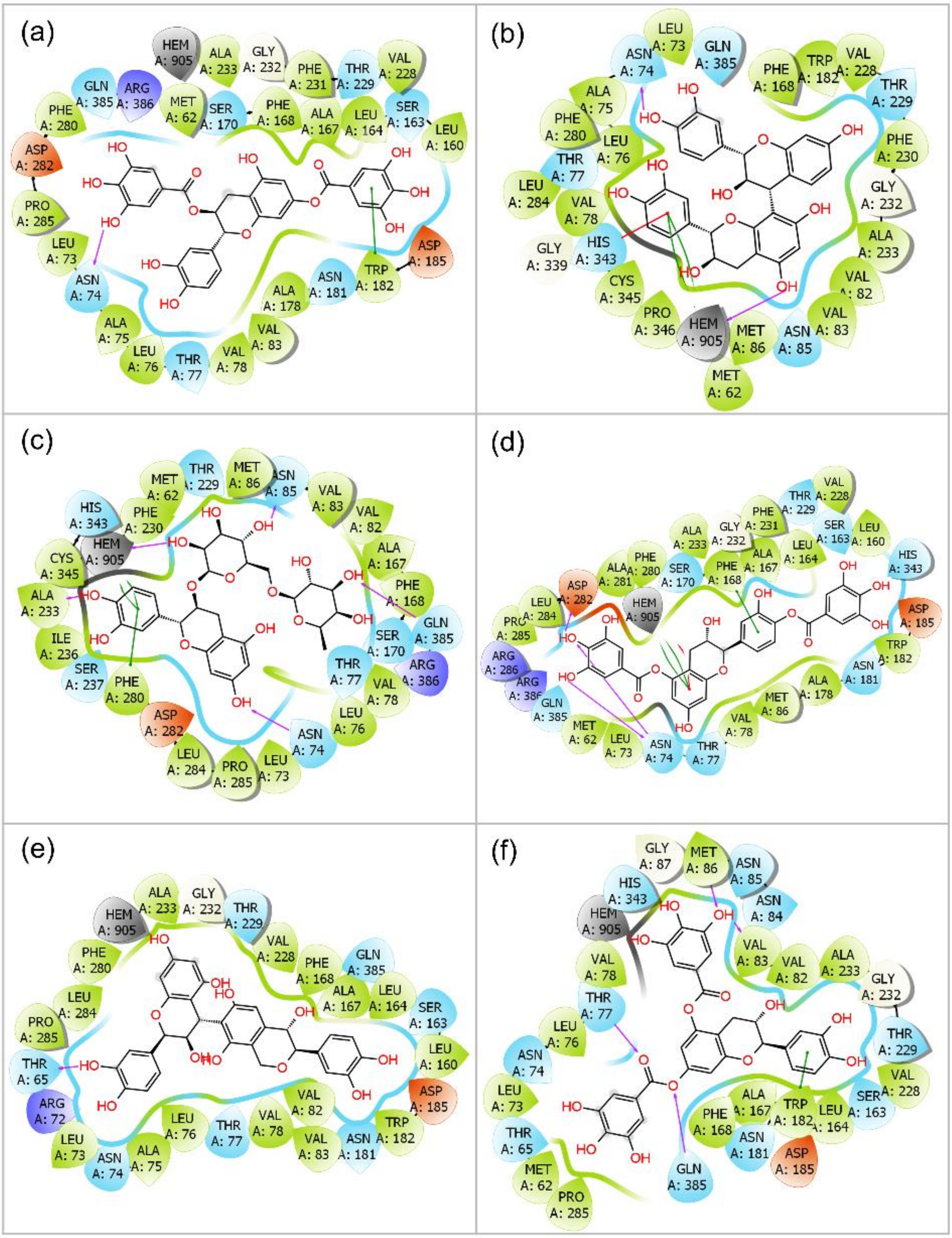

Figure S1. Two-dimensional interaction plot between protein and top 6 hit compounds. (**a**) Catechin 3,7, -Di-O-Galate(14583619), (**b**) Catechin 5,7,-Di-O-Gallate(15689621), (**c**) Catechin 5,4'-Di-O-Gallate(15689620), (**d**) Epifisetinidol-(4beta->8)-Catechin(14332863), (**e**) Catechin 3-O-Rutinoside(44257079), (f)Epicatechin (4b->6)Catechin(131752345). Herein, hydrogen bond formation (pink arrows), hydrophobic (green), polar (blue), red (negative), violet (positive) and glycine (grey) interaction are also logged between the docked Mtb CYP121 and selected ligands.

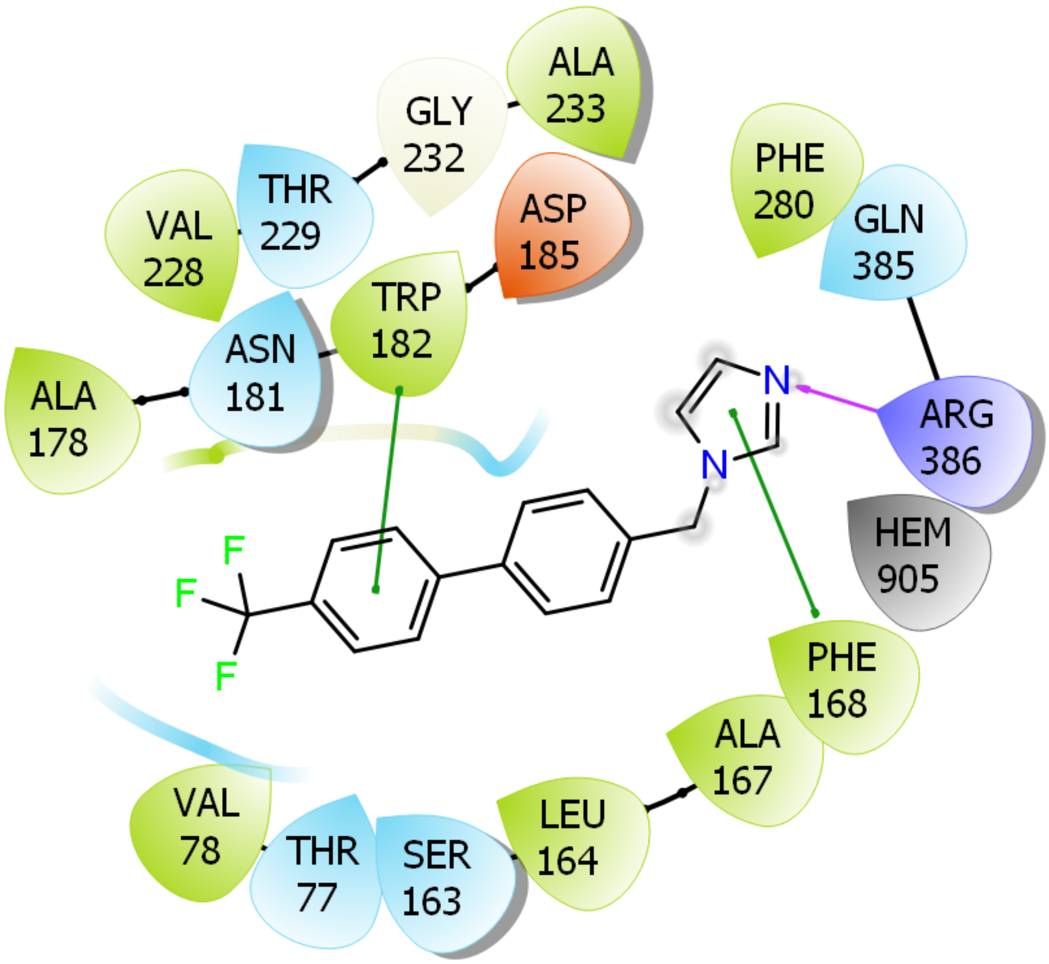

Figure S2. Interaction plot between protein and reference inhibitor. i.e., L44. Herein, hydrogen bond formation (pink arrows), hydrophobic (green), polar (blue), red (negative), violet (positive) and glycine (grey) interaction are also logged between the docked Mtb CYP121 and selected ligands.

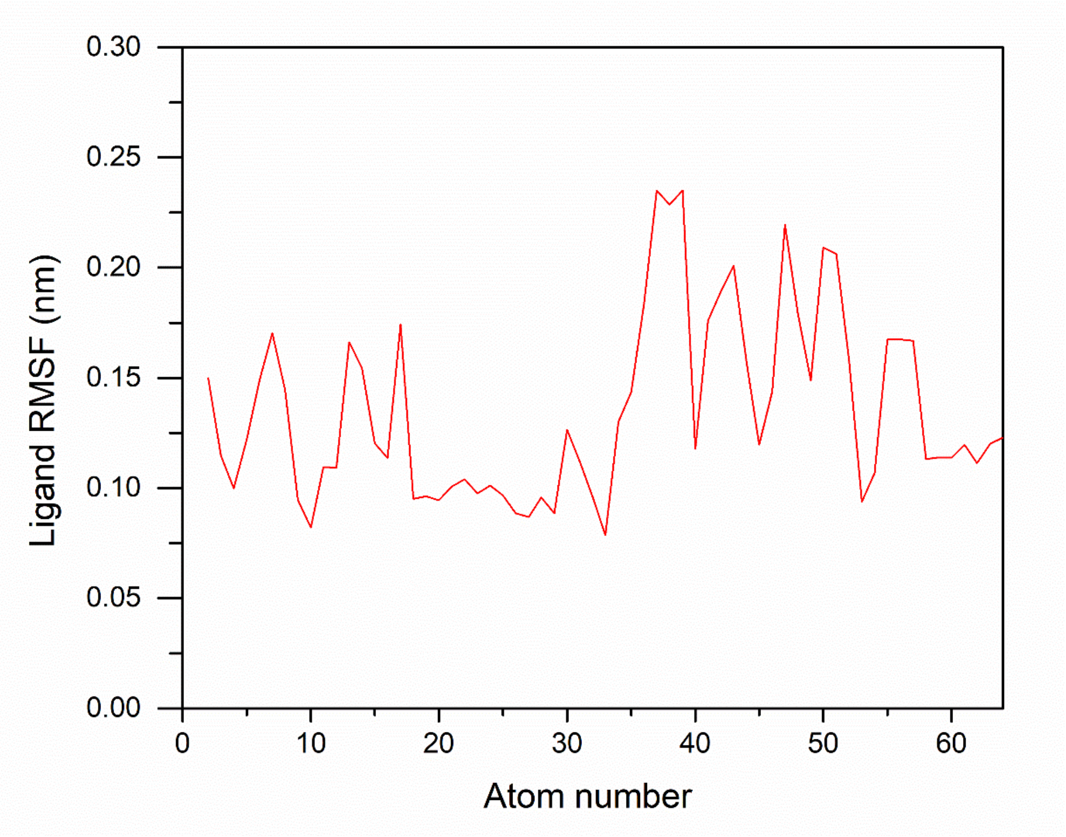

Figure S3. RMSF (root mean square fluctuation) for all atoms for reference inhibitor L44.

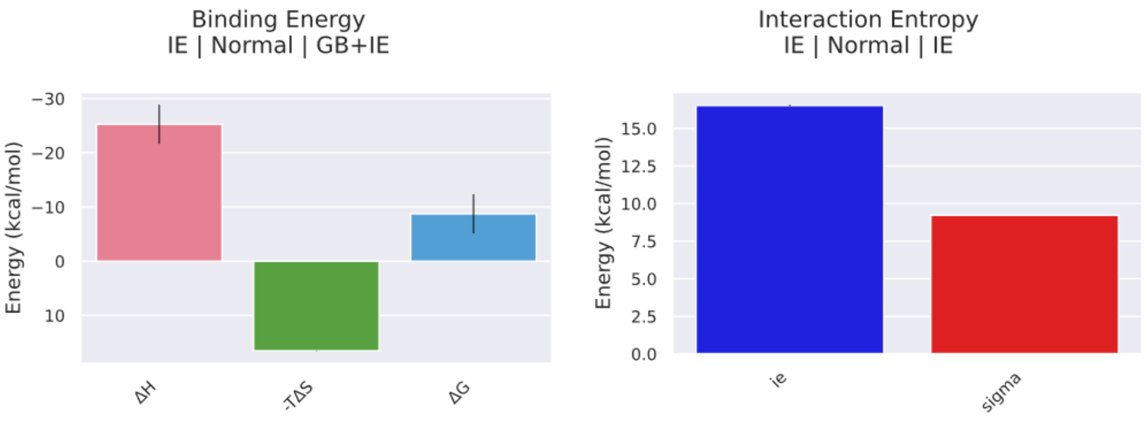

Figure S4. Binding free energy and residue decomposition score of the reference inhibitor L44 with the Mtb CYP121 protein.

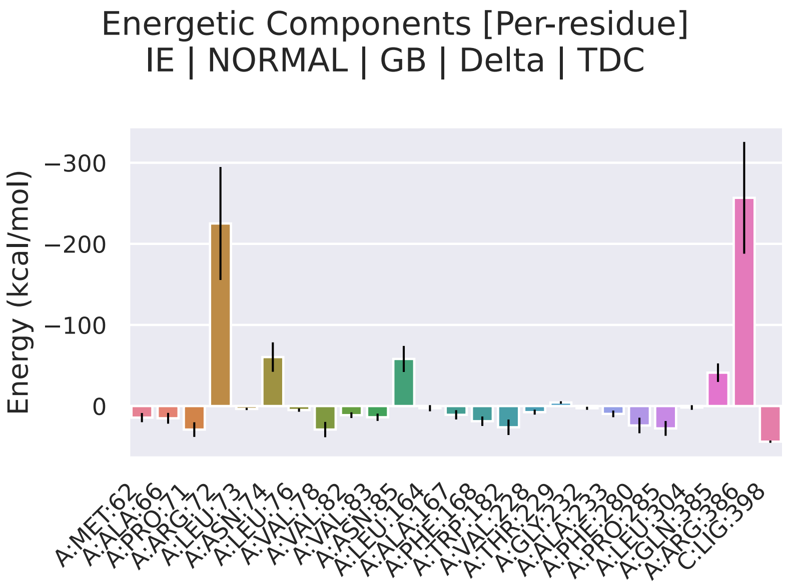

Figure S5. Free energy residue decomposition for binding site residues of the reference inhibitor L44 with the Mtb CYP121 protein.
